# Safety and efficacy of vacuum-assisted mini-percutaneous nephrolithotomy for the treatment of renal stone disease: an analysis of stone free status and postoperative infectious complications

**DOI:** 10.1590/S1677-5538.IBJU.2024.0308

**Published:** 2024-08-10

**Authors:** Kaushik P. Kolanukuduru, Osama Zaytoun, Neeraja Tillu, Asher Mandel, Zachary Dovey, Maurizio Buscarini

**Affiliations:** 1 Icahn School of Medicine at Mount Sinai Department of Urology New York NY USA Department of Urology, Icahn School of Medicine at Mount Sinai, New York, NY, USA; 2 University of Alexandria Department of Urology Alexandria Egypt Department of Urology, University of Alexandria, Alexandria, Egypt

**Keywords:** Nephrolithotomy, Percutaneous, Nephrolithiasis, Kidney Calculi

## Abstract

**Purpose:**

Vacuum-assisted mini-percutaneous nephrolithotomy (vmPCNL) is being increasingly adopted due to its faster operating times and lower incidence of postoperative infectious complications (IC), however, studies have been limited by small sample sizes. We hypothesize that vmPCNL is an efficacious treatment for renal stone disease with acceptable stone-free rates (SFR) and low incidence of IC. The objectives of this study were to measure SFR three months after surgery, determine the factors influencing SFR, and determine the rates of postoperative IC after vmPCNL.

**Materials and Methods:**

Seven hundred and sixty seven patients underwent vmPCNL for the treatment of renal stones > 20 mm at a single institution. Patients underwent postoperative computed tomography at three months to assess SFR. Postoperative fever and SIRS/Sepsis were recorded for individual patients. Multivariate logistics regression was performed to assess predictors of SFR.

**Results:**

The SFR was found to be 73.7% at three months. Stone burden (OR 0.39, 95% CI [0.33-0.46]) and age (OR 1.03, 95% CI [1.01-1.04]) emerged as statistically significant predictors of SFR on multivariate analysis. 5.5% of patients experienced postoperative fever, while 2.9% experienced SIRS/Sepsis.

**Conclusions:**

This is the largest continuous cohort of patients to undergo vmPCNL for stone disease and demonstrates that vmPCNL is safe and efficacious, with an SFR of 74% at three months. The incidence of postoperative fever and SIRS/Sepsis is 5.5% and 2.9% respectively. Further randomized studies with large sample sizes are required to ascertain the rates of these complications in comparison to conventional approaches.

## INTRODUCTION

Percutaneous nephrolithotomy (PCNL) is the surgical treatment of choice for patients with renal stones > 20 mm or lower pole stones > 10 mm (1). Mini-PCNL (mPCNL) involves the use of a miniature endoscope passed through a percutaneous tract (14 – 22 Fr) to access the renal collecting system to perform lithotripsy (2). mPCNL has similar efficacy to traditional PCNL approaches, with a superior safety profile and a reduced need for transfusion after surgery (3, 4). mPCNL has also been shown to be superior to retrograde intrarenal surgery (RIRS) in the context of postoperative stone-free rate (SFR), with similar rates of postoperative complications (5). However, a potential disadvantage to mPCNL is the longer operating times and the increased intrarenal pressures (IRP) (6).

Vacuum-assisted mPCNL (vmPCNL) is being increasingly adopted due to its faster operating times, high SFR, and low incidence of complications. The lower IRP during vmPCNL prevents excessive pyelic-venous backflow and renal pelvis damage (7).

Studies on the outcomes of vmPCNL have been limited by small sample sizes, with limited evidence on SFR and infectious complications (IC). We hypothesize that vmPCNL has acceptable SFR with a low incidence of IC and sought to determine SFR and IC rate in a contemporary cohort of patients. To our knowledge, this study represents the largest continuous cohort of patients to undergo vmPCNL for renal stone disease.

## MATERIALS AND METHODS

### Study setting, design, and participants

This prospective study was performed after obtaining institutional review board approval at the institution where the study took place (IRB ITCBM44678/12012). Between 2016 and 2022, patients with renal stone disease were offered vmPCNL for definitive surgical treatment after a shared decision-making process. After clinical examination, patients underwent routine preoperative assessments including contrast/non-contrast computerized tomography (CT), renal ultrasound (RUS), preoperative creatinine, urinalysis, and urine culture. Patients with suspicion of urinary tract obstruction or infection were stented prior to surgery. All procedures were performed by a single surgeon (MB).

### Surgical Technique

Following anesthetic induction and routine ureteral occlusion balloon placement (Boston Scientific™), patients were placed in either prone or supine position depending on individual patient characteristics (such as patient BMI, cardiorespiratory status, and high-risk for anesthetic complications), stone location, and the surgeon's decision on a case-to-case basis. Percutaneous renal access was obtained with an 18-gauge diamond-tipped needle on the appropriate calyx using standard fluoroscopic guidance. The surgical equipment used included the 12F mini-nephroscope (MIP, Karl Storz™) and the 16F ClearPetra® vacuum suction sheath (Well Lead Medical Co.™). A holmium laser was used to perform stone fragmentation and dusting (Ho:YAG laser, Lumenis/Boston Scientific™, 550 µm fiber, 100 W). Irrigation was performed with a normal saline bag placed 1.5 meters above the site of nephroscope insertion. After introducing the nephroscope and suction sheath, the negative pressure was switched on to ensure a suction effect. The vacuum pressure was set at 200 mm Hg. Stone clearance was performed until no stones could be visualized by the surgeon. Basketing was employed in select cases where the fragments were only reachable with the nephroscope. At the end of the procedure, all calyces were routinely inspected with a flexible scope passed through the sheath. An antegrade double-J stent was placed after surgery at the surgeon's discretion, which was removed between one and two weeks after surgery. All procedures were performed under fluoroscopic guidance. In cases where the total stone burden was high due to stones present in different anatomical locations of the kidney, a multistage approach was used to decrease the operative time of any single procedure, thus minimizing patient risk (42 patients [5.5%]). The surgical technique was standardized across all cases to maintain consistency in patient care.

### Data Variables and Study Measures

Preoperative variables collected included age, sex, body mass index (BMI), preoperative creatinine, and preoperative stent placement. Stone-specific characteristics like laterality, location, stone burden (mm), and stone density (measured in Hounsfield Units, HU) were also collected simultaneously. Operative characteristics included for the analysis were operative time (induction of anesthesia to end of surgery), lithotripsy time, position of vmPCNL, use of basket, stent placement after surgery, and duration of postoperative stay. Postoperative variables analyzed included postoperative creatinine, postoperative complications within 90 days (if any), postoperative fever (within 48 hours), and SIRS/Sepsis. We defined SIRS as the presence of two or more of the following: body temperature >38°C or <36°C, white blood cell count >12 × 109, or heart rate > 90 beats/minute. Patients with SIRS were diagnosed with sepsis if they also had a positive blood culture. Finally, we measured the stone-free rate (SFR) after vmPCNL, defined as the absence of any residual fragments on postoperative CT scan three months after surgery.

### Statistical analysis

Means with standard deviations were measured for continuous variables, while categorical variables were reported as absolute numbers and percentages. Logistic regression analysis was initially performed on relevant perioperative variables to determine predictors of SFR. Subsequent to this, a multivariate analysis was performed using those variables that demonstrated statistical significance (p <0.05). Odds ratios (OR) were calculated for variables in the multivariate model along with 95% confidence intervals (CI). All analysis was performed on R programming software version 4.3.3.

## RESULTS

### Baseline, Stone and Operative Characteristics

Seven hundred and sixty seven patients underwent vmPCNL for the treatment of renal stones. 57% of patients were male, while 43% were female. The mean age and BMI of the group were 49.9 ± 14.61 years and 29.7 ± 6.05 kg/m^2^ respectively. The mean preoperative creatinine was 1.41 ± 0.36 gm/dL. 50.1% of patients had a stent placed preoperatively due to suspicion of infection or urinary tract obstruction. 63.6% and 36.4% of patients had left-sided and right-sided renal stone disease respectively, with 53.6% of patients having lower pole stone disease. The mean stone burden for this group was 32.4 ± 15.6 mm. 22% of patients had high-density stones as measured by preoperative CT (HU > 950). The mean operative and lithotripsy time was 117.6 ± 43.4 and 68.9 ± 38.3 minutes respectively. 77.1% of vmPCNL were performed in prone position. Intraoperative basketing was performed in 7.95% of cases due to inadequate stone clearance using the suction evacuation alone. Following surgery, a stent was left in place in 67.5% of procedures. 96.3% of patients were admitted to the hospital overnight (Table-1).

**Table 1:** Baseline, stone, and operative characteristics of patients undergoing vmPCNL (vacuum-assisted mini-percutaneous nephrolithotomy).

PARAMETER			RESULT
Number of patients, n			767
**Sex, n (%):**			
	Male		437 (57%)
	Female		330 (43%)
Age, mean ± SD			49.9 ± 14.61
Body Mass Index, mean ± SD			29.71 ± 6.05
Preoperative Creatinine (gm/dL), mean ± SD			1.41 ± 0.36
Preoperative Stent Placed, n (%)			385 (50.1%)
**Laterality, n (%):**			
	Right		279 (36.4%)
	Left		488 (63.6%)
**Stone Location, n (%):**			
	Upper Pole and Pelvis		356 (46.4%)
	Lower Pole		411 (53.6%)
Stone burden (mm), mean ± SD			32.4 ± 15.6
**Stone Density, Hounsfield Units (HU), n (%)**			
	< 600		298 (38.9%)
	600 − 950		300 (39.1%)
	>950		169 (22%)
Operative time (minutes), mean ± SD			117.6 ± 43.4
Prone position, mean ± SD			131.5 ± 22.1
Supine position, mean ± SD			105 ± 43.7
Lithotripsy time (minutes), mean ± SD			68.9 ± 38.3
**Position, n (%):**			
	Prone		591 (77.1%)
	Supine		176 (22.9%)
Use of basket intraoperatively, n (%)			61 (7.95%)
Intraoperative stent placement, n (%)			518 (67.5%)
**Postoperative stay, n (%):**			
		< 1 day	28 (3.7%)
		≥ 1 day	739 (96.3%)

### Postoperative complications and outcomes after vmPCNL

The mean postoperative creatinine in this cohort was 1.18 ± 0.33 gm/dL, thus resulting in a mean creatinine change of −0.23 ± 0.49 gm/dL (postoperative - preoperative). 12.9% of patients experienced at least one complication within 90 days after surgery. Urinoma was noted after surgery in 1.4% of patients, while 2.3% of patients required transfusion after surgery. 3.4% and 1.4% of all patients experienced Clavien-Dindo 3 and Clavien-Dindo 4 complications respectively. The mortality rate in this study was 0.26%. At three months, 73.7% of all patients were stone-free after vmPCNL (Table-2).

**Table 2:** Postoperative characteristics and complications of patients undergoing vmPCNL (vacuum-assisted mini-percutaneous nephrolithotomy).

PARAMETER		RESULT
Postoperative Creatinine (gm/dL), mean ± SD		1.18 ± 0.33
Change in Creatinine (gm/dL), mean ± SD (postoperative - preoperative)		-0.23 ± 0.49
Total Number of postoperative complications, n (%)		99 (12.9%)
Postoperative Fever (within 48 hours), n (%)		42 (5.5%)
Postoperative SIRS/Sepsis, n (%)		22 (2.9%)
Urinoma, n (%)		11 (1.43%)
Postoperative transfusion, n (%)		18 (2.3%)
**Postoperative Complications within 90 days, n (%):**		
	Clavien-Dindo 1	34 (4.4%)
	Clavien-Dindo 2	25 (3.3%)
	Clavien-Dindo 3	26 (3.4%)
	Clavien-Dindo 4	12 (1.5%)
	Clavien-Dindo 5	2 (0.26%)
Stone free at three months, n (%)		565 (73.7%)

### Predictors of Stone Free Rate at three months

On univariate analysis, age, lower pole disease, stone burden, and position of vmPCNL (supine vs. prone) showed statistically significant associations with SFR. Including these variables in a multivariate model revealed that stone burden (OR 0.39, 95% CI [0.33-0.46], p <0.001) was inversely related to SFR, while SFR increased with age (OR 1.03, 95% CI [1.01-1.04], p <0.001). Lower pole disease (OR 1.39, 95% CI [0.92-2.1], p=0.11) and position of the patient (supine vs. prone) during vmPCNL (OR 0.57, 95% CI [0.31-1.05], p=0.07) did not yield any statistical significance when controlling for confounders in the multivariate model (Table-3).

**Table 3:** Univariate and multivariate analysis of perioperative factors influencing stone-free rate at three months.

PARAMETER			UNIVARIABLE			MULTIVARIABLE	
			Stone Free at three months	Residual Stone Disease at three months	p-value	Odds Ratio (95% CI)	p-value
Age, mean ± SD			51.2 ± 15.1	46.4 ± 12.6	<0.001	1.03 (1.01 - 1.04)	<0.001
BMI, mean ± SD			29.7 ± 6.14	29.7 ± 5.81	0.96		
**Sex, n (%)**							
		Male	316 (41.1%)	121 (15.8%)	Ref.		
		Female	249 (32.5%)	81 (10.6%)	0.33		
Preoperative Creatinine, mean ± SD			1.41 ± 0.36	1.42 ± 0.39	0.69		
Preoperative Stent Placed, n (%):			279 (36.4%)	106 (13.8%)	0.45		
Lower Pole Stone, n (%):			289 (37.7%)	122 (15.9%)	0.02	1.39 (0.92 − 2.1)	0.11
Stone Burden (mm), mean ± SD			28 ± 14.8	44.9 ± 10.8	<0.001	0.39 (0.33 - 0.46)	<0.001
**Stone Density, HU, n (%)**							
	<600		211 (27.5%)	87 (11.3%)	Ref.		
	600 - 950		228 (29.7%)	72 (9.4%)	0.15		
	> 950		126 (16.4%)	43 (5.6%)	0.38		
Lithotripsy time, mean ± SD			68.42 ± 37.5	70.4 ± 40.4	0.53		
Use of basket, n (%):			61 (7.95%)	0 (0%)	0.97		
Intraoperative stent placed, n (%):			377 (49.2%)	141 (18.4%)	0.42		
Postoperative Creatinine, mean ± SD			1.18 ± 0.33	1.18 ± 0.34	0.86		
Change in creatinine (postoperative- preoperative), mean ± SD			-0.23 ± 0.48	-0.24 ± 0.49	0.87		
**Position, n (%)**							
	Prone		425 (55.4%)	166 (21.6%)	Ref.	-	
	Supine		140 (18.3%)	36 (4.7%)	0.04	0.57 (0.31 - 1.05)	0.07

## DISCUSSION

To our knowledge, this is the largest continuous series of patients to undergo vmPCNL with the ClearPetra® system for the treatment of renal stone disease. We found that 73.7% of patients were stone-free three months after surgery. Stone burden was the only clinically significant predictor of SFR in this patient cohort. While age was also identified to be a predictor of SFR after surgery, the OR for this association tended to one (OR=1.03), thus suggesting that the significance we found was only a statistical one, with little clinical relevance. Nonetheless, further studies are required to truly ascertain the role of age as a predictive factor for SFR in the setting of vmPCNL. As the study evolved, we progressed from using intermittent suction evacuation to continuous suctioning, which we felt improved intraoperative visualization without compromising stone fragmentation and clearance if a steady flow of irrigation was maintained. The use of intraoperative basketing decreased as the study progressed and the team became more comfortable with the use of the ClearPetra® system. Additionally, we employed retrograde nephroscopy to visualize renal calyces in specific cases to confirm adequate stone clearance.

We noted that approximately 13% of patients in our cohort experienced a postoperative complication; most complications were Clavien-Dindo 1 (4.4% of the whole cohort). 5.16% of patients experienced a complication ≥ Clavien-Dindo 3. 2.3% required postoperative blood transfusion. Two patients in this study died after surgery (0.26%). Both patients had multiple comorbidities prior to surgery and were of advanced age (67 and 72 years respectively). One of these patients died due to sepsis, while the other died due to anesthetic complications. This mortality rate is in concordance with previously reported mortality rates of 0.2% after PCNL (8). These findings suggest that vmPCNL may not contribute to decreased mortality or transfusion rate when compared to PCNL, and thus, may only be useful in promoting SFR and lowering the incidence of IC after surgery. It is essential to identify the risk of postoperative complications prior to surgery and tailor the treatment approach to individual cases based on the probability of postoperative complications. Many studies have explored the use of the Charlson Comorbidity Index, Guy's Stone score, S.T.O.N.E. score, and other relevant perioperative variables to generate nomograms predictive of SFR and postoperative complications after PCNL (9–12). The published data surrounding this, however, seems to be contradictory, and many studies are limited by small sample sizes with no external validation. Further studies are essential to develop preoperative predictive models to assess the probability of SFR and postoperative complications after vmPCNL.

Five.five percent of patients in this study developed fever within 48 hours of vmPCNL, while 2.9% of all patients went on to develop SIRS/Sepsis. The suction effect of the vacuum sheath plays an important role in decreasing pyelo-venous backflow by decreasing IRP (13). Fewer microbes are translocated across the pelvis into the vasculature, resulting in a decreased incidence of postoperative IC. A recent study by Marmiroli et al. showed that vacuum-assisted procedures and decreased operative time were associated with a lower risk of IC in mPCNL patients, and that 30% of patients experience some form of IC after surgery (14). This study, however, was limited by its retrospective nature and relatively small sample size. A propensity-matched analysis by Lievore et al. noted significantly lower SFR and IC rates in the vmPCNL group when compared to mPCNL (SFR: 89.4% vs. 78.8%, Infectious complications: 7.7% vs. 25%) (15). The reporting of IC across the literature is heterogeneous and is dependent on the study population, preoperative stone characteristics, PCNL technique, postoperative antibiotic protocols, and the definitions used for these complications. While the utility of preoperative antibiotics has been well established for PCNL treatment (16, 17), further studies are required to truly ascertain the role of perioperative antibiotics in the context of vmPCNL.

Other prospective cohort studies have also explored the SFR and postoperative IC of patients undergoing vmPCNL. Zanetti et al. found that 71.3% of patients were stone-free at 1 – 3 months, while 7.4% of patients experienced fever after surgery (18). Reddy et al. noted an SFR of 77.3% of patients and a Clavien-Dindo 2 postoperative IC rate of 3.6% (19). For comparison, we noted an SFR of 73.7%, and an incidence of postoperative fever and SIRS/Sepsis of 5.3% and 2.9% respectively, thus confirming the findings of these prospective studies.

To our knowledge, three randomized controlled trials (RCTs) have compared the postoperative outcomes of the ClearPetra® system for vmPCNL (Supplementary Table-1). The study by Lai et al. had 38 patients in each arm and noted that the use of vmPCNL increased SFR (94.4% in vmPCNL vs 86.8% in mPCNL) and decreased the incidence of Clavien-Dindo 2 postoperative fever (15.8% in vmPCNL vs. 21.1% in mPCNL) (20). Xu et al. explored SFR and postoperative fever rates between the two techniques in the context of staghorn calculi, with 30 patients randomized to each arm. The authors found that while vmPCNL was associated with a lower incidence of postoperative fever (6.6% vs. 20%), there was no difference in SFR at three months (21). Finally, an RCT by Liang et al. randomized 59 and 58 patients to vmPCNL and mPCNL respectively. The authors found that there was no significant difference in SFR at 30 days postoperatively. While they did note a trend of higher incidence of postoperative fever in the mPCNL group, the study had too few events to draw any meaningful conclusions from these results (22). These studies, while randomized in nature, are limited by their small numbers, and thus preclude the need for trials with larger sample sizes. The trends identified in our study may serve as a reference point for statistical powering of future RCTs.

**Supplementary Table 1:** Randomized controlled trials comparing outcomes between conventional minipercutaneous nephrolithotomy (mPCNL) and vacuum-assisted mini-percutaneous nephrolithotomy (vmPCNL).

AUTHOR (YEAR)	CHARACTERISTICS		NUMBER IN EACH GROUP		STONE SIZE (MM)		TRACT SIZE	STONE TREATMENT TIME		POSTOPERATIVE COMPLICATIONS		SFR DEFI- NITION	SFR	
	Group A	Group B	Group A	Group B	Group A	Group B		Group A	Group B	Group A	Group B		Group A	Group B
Lai et al. (2020) (20)	mPCNL	vmPCNL	38	38	20.2 ± 6.5	23.4 ± 7.3	18 Fr	70.4 ± 14.8	56.3 ± 19.8	Fever: 21.1% Trans- fusion: 2.7%	Fever: 13.2% Trans- fusion: 2.7%	Absence of residual fragments on NCCT, 30 d after surgery	86.80%	94.40%
Xu et al. (2020) (21)	mPCNL	vmPCNL	30	30	38 ± 14	42 ± 10	20 Fr	69.5 ± 29.4	54.2 ± 28.7	Grade 1: 20% Grade 2: 13.3% Grade 3: 3.3%	Grade 1: 6.6% Grade 2: 6.6% Grade 3: -	Absence of residual fragments > 4 mm on NCCT, 3 months after sur- gery	76.60%	90%
Liang et al. (2023) (22)	vmPCNL	mPCNL	59	58	27.7 ± 5.7	28.8 ± 5.5	18 Fr	26.9 ± 14.3	35.7 ± 11.8	Grade 1: 5.1% Grade 3: -	Grade 1: 10.3% Grade 3: 1.7%	Absence of residual fragments > 4 mm on NCCT, 1 month after sur- gery	96.60%	89.70%

Our study, however, has notable limitations. Firstly, given that this was a prospective single-arm study, we did not have a comparator group of patients who underwent mPCNL. Instead, we opted to focus on the surgical technique and the postoperative outcomes after vmPCNL alone. Additionally, we did not record IRP during this study and thus were not able to test the association between IRP and IC. Finally, we did not report on the antibiotic protocols used in the study as these changed over time with changes in antibiotic resistance patterns and hospital protocols at the center where the study was performed.

Despite these limitations, we believe this study is of value, as it represents the largest continuous series of patients to undergo vmPCNL. The results of this study demonstrate that vmPCNL is a safe and efficacious technique for stone clearance, with an acceptable SFR and a low incidence of postoperative infectious complications. Stone burden is a clinically meaningful predictor of SFR in this population of patients.

## CONCLUSIONS

vmPCNL is a safe and efficacious technique for stone clearance in patients with renal stone disease, due to the low incidence of serious complications, IC, and an SFR of 73.7% at three months. Stone burden is a significant predictor of SFR. 5.5% of patients experience fever after surgery, while 2.9% of patients develop SIRS/Sepsis. Further randomized studies with large sample sizes are necessary to truly ascertain the differences between vacuum-assisted and conventional approaches.
